# Stressful but Not Unhappy: A Review of the Positive Aspects of Parenting a Child with Autism Spectrum Disorder

**DOI:** 10.3390/children12010107

**Published:** 2025-01-17

**Authors:** Kenneth Curley, Robert Hughes, Yasuhiro Kotera

**Affiliations:** 1 College of Health, Psychology and Social Care, University of Derby, Derby DE22 1GB, UK; 2 School of Health Sciences, University of Nottingham, Nottingham NG7 2HA, UK

**Keywords:** parenting a child with ASD, positive parenting, positive aspects of parenting a child with ASD, positive psychology, parenting experiences, optimism, balanced reporting

## Abstract

This review aimed to identify and synthesise the evidence for the positive aspects of parenting a child with autism spectrum disorder (ASD). To date, ASD parental research has predominantly focused on the negative aspects; parents and carers are left uninformed of how to strengthen their positive mental wellbeing. Three electronic databases—PubMed, Scopus, and Web of Science —were searched for literature reporting positive aspects of parenting a child with ASD, published between January 2013 and December 2023. The PRISMA 2020 checklist was used for reporting. Two hundred and fifty-four articles were retrieved from the databases. After title/abstract screening (*n* = 213) and full-text reviews (*n* = 32), six papers were included (*n* = 6). Sixteen positive aspects of parenting a child with ASD were identified and grouped into three themes: joyful moments, journey to resilience, and social connection. These three themes are eudaimonic wellbeing constructs and often under-represented in ASD parental studies. Focusing on joyful moments, building resilience through the parenting journey, and finding support and connection with others in similar situations can support the mental wellbeing of parents and carers of children with ASD. Future research is needed to further evaluate the positive aspects of parenting a child with ASD.

## 1. Introduction

### 1.1. Autism Spectrum Disorder

Autism spectrum disorder (ASD) is a neurodevelopmental condition that significantly impacts an individual’s social interaction, communication, and behaviour patterns [1,2,3]. ASD’s impact on social interaction includes challenges in understanding and responding to social cues and difficulties in participating in conversations. ASD’s effects on communication include delayed language development and difficulty understanding nonverbal cues. ASD’s impact on behaviour involves pervasive repetitive behaviours that focus on specific interests [1,4]. The World Health Organization (WHO) [5] estimates that ASD affects one in every 270 children globally, and with each child a parent; this statistic equates to 37 million parents worldwide.

An important factor in examining the shortage of positive research on autism parenting relates to the historical view of the condition and that in the clinical and public domains. Until recently, much of the research supporting diagnosis and classification has been from a development deficit basis. This has influenced generations of clinicians and the public understanding of autism, affecting the experiences of autistic individuals and their families [6,7,8]. This lack of focus on the positive aspects of parenting could therefore be strengthened through the lens of positive psychology, a field of psychology concerned with the study of positive emotions and which emphasises strengths, wellbeing, and flourishing [9].

The term “autism” was first used by Paul Eugen Bleuler in 1911 [10], and was initially considered a symptom of schizophrenia. It was not until the 1940s, with Kanner’s study on infantile autism [11], that autism began to be recognised as a separate condition from schizophrenia. Hans Asperger’s began work on “autistic psychopathy” in 1944 [12]; however, it was not until Lorna Wing’s proposal of Asperger’s syndrome [13] that it gained attention in English-speaking countries [14].

Early to mid-twentieth-century theories, influenced by Freudian psychoanalytic ideas, suggested that autism stemmed from emotional or psychological factors rather than biological or neurological ones [15]. Kanner’s study [11] implied that parents of autistic children were emotionally distant, contributing to the “refrigerator mother” theory [16]. This theory persisted in clinical and public discourse until research in the 1960s began to emphasise biological and neurological causes of autism [17]. Autism was officially categorised in the DSM-III in 1980 [14] and was consolidated under the term autism spectrum disorder in the DSM-5 in 2013 [14].

### 1.2. Parenting a Child with ASD

Parenting a child with ASD is believed to be meaningful and rewarding [18]; however, there is a significant body of research focusing solely on the challenges faced by parents of children with ASD [19], including child maladaptive behaviour, a key factor associated with elevated stress levels of parents [20]; communication difficulties, arising when trying to understand needs in non-verbal communication [21]; anger and frustration arising from experiencing social stigma in public places; and isolation, often the result of a lack of support from either family, friends, or the community [22]. While it is essential to explore these challenges, there is less research on the rewards of parenting a child with ASD [23], such as the positive aspects, the joy in watching a child grow in innocence and wonder, and seeing their excitement when experiencing new fun things or activities [24,25]. One systematic review concluded that there is an existing body of evidence about the positive aspects of caring, but little on the positive aspects of parenting children with ASD [23]. Beighton [23] explained how positive aspects of parenting were attributed to theories relating to coping, adaptation, and growth associated with adversity, and that positive change does not mean the absence of distress. The positive aspects of parenting a child with ASD can be considered as a parent’s positive perception of an experience, situation, or outcome; these include joy and satisfaction in parenting, perceived personal growth, or bonding between parent and child [24].

### 1.3. Positive Aspects of Parenting

Whilst exploring challenges associated with parenting a child with ASD is undoubtedly an important topic, investigating the positive aspects associated with the parenting experience is equally necessary. One study concluded that parents of children with ASD experience increased personal growth, confidence, and meaning-focused coping strategies, which assist in adapting to stressful experiences [26]. Meaning-focused coping is the response that reduces physical, emotional, and psychological stress, and emphasises the cognitive and emotional elements of stress, focusing on stressor evaluation and the identification of coping opportunities [26]. An example of this might be reframing challenges as an opportunity for growth or finding new meaning by participating in support groups. Therefore, the implications of research that explores this topic may help create awareness of the positive aspects of parenting a child with ASD, promote the personal growth of parents, increase their confidence, and strengthen meaning-focused coping strategies.

### 1.4. Problem Statement

Although there is a great deal of research on the challenges and stresses that parents of autistic children face, there is less research on the positive aspects of parenting an autistic child [4]. This is a problem because in order to maintain high levels of mental wellbeing, we need to understand both positive and negative mental health [27,28]. Exploring this deeper may assist in celebrating the joys that are not often highlighted enough in research. Therefore, our research question was “What are the positive aspects of parenting a child with ASD?”.

## 2. Materials and Methods

### 2.1. Search Strategy

Three electronic databases—PubMed, Scopus, and Web of Science—were searched for relevant literature; the search terms used were the keywords provided by this review: parenting a child with ASD, positive parenting, positive aspects of parenting a child with ASD, positive psychology, parenting experiences, optimism, and balanced reporting. PubMed was considered suitable as it offers extensive coverage of scholarly journals and is believed to be suitable for this under-researched topic. As Scopus consists of a large body of social science literature, it was chosen for this review. Web of Science was chosen for its comprehensive coverage of peer-reviewed literature across various disciplines. The search was conducted on 15 January 2024 by using the electronic reference manager Endnote [29]. Searches were conducted by K.C., and the search results at each step were reviewed by R.H.

### 2.2. Inclusion/Exclusion Criteria

The inclusion/exclusion criteria for this review were established using an extended version of the PEO (population, exposure, and outcomes) framework [30] (Table 1), a framework used for literature searches to find evidence.

### 2.3. Quality Assurance

The PRISMA 2020 checklist was utilised to ensure clear and coherent reporting, and the PRISMA flowchart was used to display the search strategy process [31]. To assess the quality of included studies, the critical appraisal skills programme (CASP) checklist was used [32]. The lead author, K.C., provided an initial assessment, which was then reviewed by the rest of the research team (“level of quality” in Table 2; full CASP table found in the Appendix A).

### 2.4. Evidence Synthesis

Positive aspects of parenting a child with ASD were found in the literature included in this review and were thematically synthesised, following Kotera et al.’s [33] application of a thematic synthesis process. To structure this synthesis, we specifically adopted Thomas and Harden’s [34] three-step approach, which involved coding, the development of descriptive themes, and the generation of analytical themes of interpretative constructs and explanations.

**Table 2:** Data abstraction table.

Author/Year	Level of Quality	Population	Sample Size	Purpose	Theoretical/ Conceptual Framework	Exposure and Follow-Up	Design and Type of Research	Instruments Used	Analysis and Results	Conclusions	Implications
[35]	High	Mothers of children with ASD	*n* = 11	Examine the positive aspects of mothering a child with ASD	Positive psychology	Three interviews over 4–6 weeks. Duration: 1–2 h each	Qualitative study/interpretive phenomenology	Auchenbach child behaviour checklist (CBC). Semistructured interviews	Interpretive phenomenological analysis produced three themes: silver lining, transformation of mothers, and appreciation of little things	Parenting a child with ASD can result in developing a positive outlook, increased resilience, and a newfound sense of empowerment. Mothers also obtain profound joy in witnessing their child’s milestones	Inform HCP on the positive aspects of mothering a child with ASD, the value of creating opportunities for strengthening resilience, and how optimism alleviates the focus on child limitations
[36]	Mid	Mothers of children with ASD	*n* = 8	Explore the experiences of mothers raising a child with ASD	Double ABCX model/Seligman’s PERMA framework	A single interview. Duration: 60–90 min	Qualitative study/interpretive phenomenology	Semistructured interviews	The data analysed produced three themes: problem realisation within the context: learning to speak autism in Malaysia; we live with autism; and resilient overcoming: climbing Mount Kinabalu	Seligman’s PERMA framework may apply to understanding parental wellbeing. Intrapersonal processes: acceptance, proactive mindset, character growth, spiritual coping, and interpersonal processes: positive relationships with peers and experts can empower and develop wellbeing for parents of children with ASD	Inform HCPs on tailoring approaches to align with cultural sensitivities, considering incorporating intrapersonal strategies into intervention programmes and the need for including fathers in supporting interventions. Inform policymakers to improve education facilities and teacher training, provide financial support, reduce treatment costs, and access diagnostic resources. Promote awareness of the experiences of mothers of children with ASD
[37]	High	Parents of children with ASD in China	*n* = 20	Explore the identity of parents of children with ASD	The ethics of care theory	Two interviews: round 1 was in 2014, and round 2 was in 2017. Duration: 2–6 h each	Qualitative study/thematic analysis	In-depth interviews	Data were analysed using thematic analysis, which produced three themes: recognition, rights, and redistribution of care for children with ASD	Despite their challenges, parents can utilise strategies, such as cultivating a positive self-perception. Some parents demonstrate inner strength, leading to active engagement in support networks and self-advocacy	Inform HCPs and policymakers on the importance of the recognition of parents’ caring efforts, the need to reduce social stigma surrounding parents of children with ASD, and increasing social awareness of challenges faced by parents. HCPs should focus on the strengths and achievements of parents of children with ASD and reinforce self-perception to foster resilience and confidence among parents
[38]	Mid	Fathers of children with ASD in Ireland	*n* = 9	Examine the perceptions of fathering a child with Asperger’s syndrome, a subtype of autism spectrum disorder	Husserlian phenomenology	A single interview. Duration: 60–90 min	Qualitative study/phenomenological approach	Semistructured interviews guided by open-ended questions	Colaizzi’s six-step method of phenomenological analysis produced three themes: the journey from awareness to a diagnosis, living with a child with Asperger’s syndrome, and the impact of services	While fathering a child with Asperger’s was reported to be challenging, fathers expressed pride, joy, and a solid emotional connection with a child. One possible reason for this is a positive demeanour when faced with adversity	Inform HCPs on recognising the role that fathers play in the caring process, supporting and encouraging fathers of children with ASD to cultivate a positive perspective for fostering resilience in times of adversity
**Author/Year**	**CASP**	**Population**	**Sample Size**	**Purpose**	**Theoretical/** **Conceptual** **Framework**	**Exposure and Follow-up**	**Design and Type of Research**	**Instruments Used**	**Analysis and** **Results**	**Conclusions**	**Implications**
[39]	High	Parents of children with ASD	*n* = 22	Explore the experiences of parents of children with ASD to understand relationship satisfaction between parents	Family resilience framework	A single interview. Duration: 1–2 h each	Qualitative study/phenomenological approach	The couples satisfaction index (CSI). Semistructured interviews	Data were analysed using NVivo 11 software to assist the thematic analysis. Three themes emerged: shared beliefs, teamwork, and shared experiences	While parenting children with ASD can be challenging, resilience through collective commitment can be attained with shared beliefs, teamwork, and shared experiences	Inform HCPs on promoting acceptance, positive reframing, and shared beliefs; emphasise the importance of effective communication and joint problem-solving activities among parents to enhance parents’ ability to navigate complexities associated with parenting children with ASD
[40]	High	Parents of children with ASD in China	*n* = 5	Examine the resilience of parents with children with ASD. This study recruited highly resilient parents using a resilience scale to capture data that assist mothers in navigating adversity	Resilience theory and positive psychology	Three interviews. Duration: 1–2 h each	Qualitative design/case study method	Connor–Davidson resilience scale (CD-RISC)/semistructured interviews	Data were analysed using NVivo 8.0. Open coding and the constant comparison method followed. Three themes were produced, highlighting the interactions among three social systems: micro–mezzo–macro	Building a positive relationship with oneself by cultivating moral principles and introspection, engaging in social interaction, and adopting a mindset of accepting a worst-result scenario may build resilience for parents of children with ASD	Inform HCPs on the strength-based approach to parenting a child with ASD and how that may empower parents; recognise parents exist in social systems and therefore encourage and guide social interactions that foster resilience; and the value of focusing on optimistic theoretical interventions

## 3. Results

**Figure 1:**
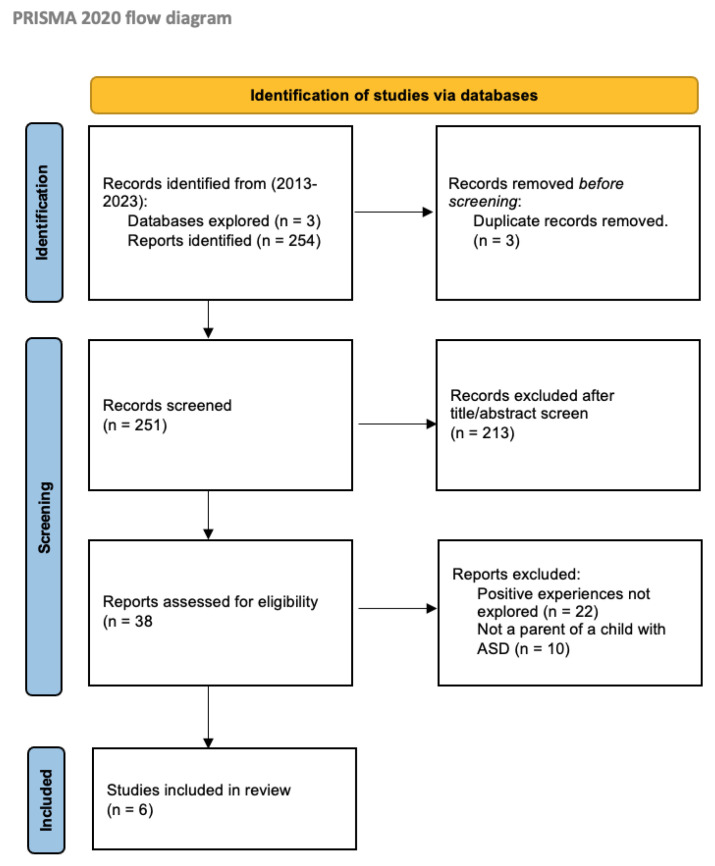
PRISMA 2020 diagram of the search strategy.

All six included studies were qualitative studies (Figure 1). A total of 16 positive aspects of parenting a child with ASD were identified, forming three themes: joyful moments (five positive aspects), journey to resilience (six positive aspects), and social connection (five positive aspects). Specific positive aspects associated with the three themes are summarised in Table 3.

The five positive aspects identified in joyful moments symbolise the joys parents experienced when they witnessed milestones and were perceived as joyful and rewarding [38]. The six positive aspects in journey to resilience symbolise how parents observe, discover, or cultivate inner strength and optimism, a positive aspect of parenting [39]. The five positive aspects of social connection signify how social interaction and support networks have a positive therapeutic effect [40]. The critical appraisal and quality notes can be found in Table 2, the data abstraction table, consisting of data extracted from the articles included in the review, to compare and summarise critical data in an efficient presentation.

Joyful moments: This theme highlights parents’ experiences of joy, pride, and happiness when raising their child; these were related to development milestones, expressions of affection, and unique aspects of a child’s personality. For example, Bultas and Pohlman [35] highlight that parenting a child with ASD can result in developing a positive outlook, increased resilience, and a newfound sense of empowerment. Mothers also obtain profound joy in witnessing their child’s milestones. Journey to resilience: This theme explains the process some parents experience in developing inner strength and optimism. Sim et al. [39] detail how parents developed resilience with shared beliefs, teamwork, and shared experiences. Social connection: This theme highlights how parents found social interaction essential for sharing experiences with others in similar situations [40].

### Characteristics of Included Studies

Zhao and Wangqian [40] conducted a qualitative case study to examine the resilience of parents of children with ASD in China. Highly resilient parents (*n* = 5) were recruited after scoring high on a resilience scale, and researchers would then determine specific tools and strategies that assisted them in navigating through adversity. The study consisted of three interviews of one to two hours, and the data were later analysed by using NVivo 8 software for coding [41]. Three themes were produced on the micro, mezzo, and macro systems: building a positive relationship with oneself by cultivating moral principles and introspection, engaging in social interaction, and adopting a mindset of accepting a worst-result scenario to build resilience for parents of children with ASD [40].

Bultas and Pohalman [35] conducted a qualitative interpretive phenomenological study examining the positive aspects of mothering a child with ASD. Eleven mothers of various ethnicities participated in three semistructured interviews over four weeks followed by a two-week follow-up. Three themes emerged from the data analysis: silver lining, transformation of mothers, and appreciation of little things [35]. The findings concluded that parenting a child with ASD can result in some mothers developing a positive outlook, increased resilience, and a found sense of empowerment. Mothers also enjoy witnessing their child’s milestones [35].

O’Halloran et al. [38] conducted a qualitative study to investigate the perceptions of fathering a child with Asperger’s syndrome, a subtype of ASD. Nine fathers in Ireland participated in a single semistructured interview of 60–90 min. Colaizzi’s six-step method of phenomenological analysis [42] was employed, producing three themes: the journey from awareness to a diagnosis, living with a child with Asperger’s syndrome, and the impact of services [38]. While fathering a child with Asperger’s syndrome was reported as challenging, fathers expressed pride, joy, and a solid emotional connection with the child—one possible reason for this is a positive demeanour when faced with adversity [38].

Liu and Fisher [37] conducted a qualitative study to explore the identity of parents of children with ASD in China. Twenty parents participated in two in-depth interviews three years apart, which lasted between two and six hours each. Thematic analysis was used, which produced three themes: recognition, rights, and the redistribution of caring for children with ASD [37]. Despite challenges, parents utilised strategies, such as cultivating a positive self-perception. Some parents demonstrated inner strength, leading to active engagement with support networks and self-advocacy [37].

Sim et al. [39] conducted a qualitative phenomenological study evaluating parents’ experiences of children with ASD to understand relationship satisfaction between parents. Twenty-two parents participated in semistructured interviews, which lasted 60 to 120 min. Using NVivo 11 to analyse the data collected [41], three themes emerged: shared belief, teamwork, and shared experiences [39]. While parenting children with ASD can be challenging, resilience through collective commitment can be attained with shared beliefs, teamwork, and shared experiences [39].

Ilias et al. [36] conducted an interpretative phenomenological study exploring the experiences and wellbeing of mothers raising children with ASD in Malaysia. Eight parents participated in a single semistructured interview of 60 min to discuss their experiences of parenting. The data analysed produced three themes: problem realisation within the context: learning to spell autism in Malaysia; living with autism; and resilient overcoming: climbing Mount Kinabalu. Researchers concluded that Seligman’s PERMA framework [43] might apply to understanding parental wellbeing [36], consisting of intrapersonal processes: acceptance, a proactive mindset, character growth, spiritual coping, interpersonal processes, and positive relationships with peers and experts can empower and develop the wellbeing of parents of children with ASD [36].

In total, 16 positive aspects were reported from the six included studies. The 16 positive aspects were then categorised into three themes corresponding to our research question. The positive aspects of parenting a child with ASD are (a) joyful moments: despite the challenges, parents experienced immense joy and happiness witnessing their child’s achievements and loving embrace, and this was believed to contribute to parent resilience; (b) journey to resilience showed how parents reported a sense of personal growth and often the natural development of resilience to the challenges of parenting a child with ASD; and (c) social connection illustrated the importance of support networks and their positive impact on parents in similar situations. Many positive aspects were found, and many of them are relevant to eudaimonic wellbeing constructs.

## 4. Discussion

This review aimed to identify the positive aspects of parenting an ASD child. The six articles provided 16 positive aspects of parenting a child with ASD, grouped into three themes: joyful moments (five positive aspects), journey to resilience (six positive aspects), and social connection (five positive aspects).

Findings from the appraisal of the five positive aspects of joyful moments show that despite the challenges that parents of children with ASD report, including high parenting stress [44], parents still experience immense joy and satisfaction in witnessing their child’s achievements. Studies exploring the positive aspects of parenting typical children spotlighted experiences of unconditional love between parent and child, joy in watching a child learn and grow, and feelings of pride when seeing a child accomplish important milestones [45]. This experience is believed to contribute to resilience; thinking positively about parenting a child is argued to reduce stress and improve wellbeing, and this promotes strong and close relationships between parent and child [46], supporting perceived coping mechanisms, and increasing a sense of resilience [47]. Additionally, holding an optimistic viewpoint can increase parents’ wellbeing and quality of life [48,49]. Wellbeing and quality of life are regarded as positive mental health constructs, leading to resilience in stressful situations [50,51]. Joyful moments are a positive psychology construct protecting the mental health of parents of ASD children.

The study also found that journey to resilience revealed that some parents of children with ASD often appear to naturally develop an optimism for life, which assists them as a coping strategy against stressors so often reported in research. This finding is supported by a meta-analysis reviewing the protective effects of positive expectancies; how hope, self-efficacy, and optimism protect against the development of post-traumatic stress disorder by strengthening resilience [52]. Therefore, cultivating an optimistic view is a key aspect of fostering resilience. Moreover, positive psychology studies found that compassion is a strong associative to resilience [53,54].

In addition, some parents have sought to cultivate moral principles and virtues that focus more on psychological growth [55], developing inner strengths such as courage, hope, perseverance, and optimism, instead of placing pressure on their children to fit social norms or expectations [9]. A systematic review and meta-analysis investigating the effectiveness of positive psychology interventions, including character strengths, found extensive evidence to support this finding and their effectiveness in reducing stress and promoting wellbeing [56].

Social connection showed how sharing experiences and insights and building strong support networks among parents of children with ASD created hope, new ways of seeing the situation, and a sense of connectedness with others, which increased wellbeing [57]. This finding is consistent with a large body of research showing that social connectedness is an essential determinant of health and wellbeing [58]. A sense of belonging, support, and meaning is thought to help individuals deal and cope with stress as well as difficult emotions.

Those three themes are relevant to eudaimonic wellbeing as subjective experiences associated with living a virtuous life and a parent’s search for excellence [59]. Eudaimonic wellbeing theory is a theory that posits a person’s true happiness and fulfilment stem from living a life of purpose, virtue, and meaning [59]. The positive aspects identified in this review provide insight into the unique joys, coping strategies, and opportunities for growth that can come with parenting a child with ASD. Exploring the positive aspects of this review, parents of children with ASD may find opportunities for moments of joy, build resilience through their journey of parenting, and find support from and connection with others in similar situations.

### 4.1. Limitations

A significant limitation regarding the results of this review is that the positive aspects of parenting a child with ASD may not apply to all parents and that individuals react and respond differently to similar experiences [60]; therefore, while evaluating the potential of positive strategies, it is essential to acknowledge that not every parent may experience the benefits observed [61]. Another limitation of this review is central to the very rationale for investigating this topic: the amount of literature was small, which is why the initial dataset search only retrieved 254 studies. Therefore, this limited body of knowledge is a significant limitation for gathering data on this topic. Further research in this area should explore how parent demographics influence the experiences of positive aspects.

A third limitation to note is that the experiences and perspectives of parents in the included studies may not capture the totality of experiences and that there may be other factors that were not captured that contributed to the positive aspects of parents, which could have influenced their optimism or level of resilience, such as an individual’s demeanour or the level of expertise the individual had access to. Nevertheless, the experiences described offer valuable insights into the positive aspects of parenting a child with ASD. Finally, there may be some research team bias, because all authors are parents of a child with ASD.

### 4.2. Implications and Future Research

The findings outlined in this review provide valuable implications for healthcare professionals (HCPs), parents, policymakers, and researchers, and emphasise the benefits of focusing on the positive aspects of parenting a child with ASD while highlighting the advantages of integrating strength-based approaches, resilience cultivation, and creating social networks. Joyful moments can inform HCPs to encourage parents to celebrate their children’s milestones, as these moments can contribute to a sense of wellbeing and promote positive parenting experiences. Journey to resilience can inform HCPs, parents, and researchers about the benefits of cultivating and holding an optimistic view that seeks out the silver linings in experiences and situations, as doing so not only strengthens one’s resilience but also reduces stress and promotes wellbeing. Social connection can inform HCPs, parents, and researchers of the importance of building strong support networks, creating a sense of belonging and support, and a space to make sense of complex and often very stressful experiences, further increasing a sense of wellbeing and quality of life.

Future research may investigate how strength-based parenting and virtue-based approaches might impact parenting a child with ASD. Another possible direction for research might investigate how integrating resilience, optimism, and social factors into an intervention impacts the parenting experiences when parenting a child with ASD or learning disabilities. This review can also serve as a call to researchers to provide balanced reporting and broaden the scope of their investigations to include the positive aspects of parenting a child with ASD.

## 5. Conclusions

The purpose of this review was (1) to broaden the body of literature on the positive aspects of parenting a child with ASD, (2) to dispel possible negative stereotypes associated with parenting a child with ASD, (3) to promote the joys of parenting a child with ASD, and (4) to call on researchers to broaden their investigations to include the positive aspects of parenting a child with ASD in their reports. By critically appraising and analysing the six included articles, 16 positive aspects of parenting a child with ASD were found and organised into three groups. Joyful moments: despite the challenges, parents experienced immense joy and happiness when witnessing their child’s achievements and loving embrace, and this was believed to contribute to parent resilience. Journey to resilience showed how parents reported a sense of personal growth and often the natural development of resilience to the challenges of parenting a child with ASD. Social connection illustrated the importance of support networks and their positive impact on parents in similar situations. Focusing on joyful moments, building resilience through the parenting journey, and finding support and connection with others in similar situations were suggested to strengthen eudaimonic wellbeing, leading to a greater sense of purpose and meaning. This review found that parenting a child with ASD can bring immense joy and happiness to parents while also contributing to resilience, personal growth, and social connection.

## Figures and Tables

**Table 1:** Extended PEO framework for this review.

Review Question	What Are the Positive Aspects of Parenting a Child with ASD That Are Often Overlooked in Research?	
	**Inclusion Criteria**	**Exclusion Criteria**
Population	Parenting a child with ASD	Parenting a child without ASD
Exposure	Articles that challenge prevailing reports that parenting a child with ASD is often characterised by unhappiness and suffering	Articles that only report stress and unhappiness when parenting a child with ASD
Outcomes	Articles that provide insight into positive aspects of parenting a child with ASD and their consequences	Overemphasis on the negative aspects of parenting, which leads to increased stress, anxiety, and feelings of inadequacy
Study design	Empirical research designs that examine the positive aspects of parenting a child with ASD	Research studies that exclusively focus on challenges and difficulties without considering positive aspects of the experience
Other	Published in a peer-reviewed journal in the English language	Non-peer-reviewed journals

**Table 3:** Three themes.

Joyful Moments	Journey to Resilience	Social Connection
Witnessing milestones	Catching the silver lining	Sharing experiences and insights
Receiving loving embrace	Cultivating moral principles	Building strong support networks
Celebrating small victories	Adopting a mindset of acceptance	Fostering resilience through social support
Appreciating child strengths	Developing inner strength	Social support can create hope
Finding joy in everyday moments	Embracing the positives in life	Recognising that sharing creates new perspectives
	Realising the value of optimism	

## Appendix A

	**Title**	**Was There a Clear Statement of the Aims** **of the Research?**	**Is a Qualitative Methodology Appropriate?**	**Was the Research Design Appropriate to** **Address the Aims of the Research?**	**Was the Recruitment Strategy Appropriate to the Aims of the Research?**	**Were the Data Collected in a Way That Addressed** **the Research Issue?**	**Has the Relationship Between the Researcher and Participants Been Adequately Considered?**	**Have Ethical Issues Been Taken into Consideration?**	**Was the Data Analysis Sufficiently Rigorous?**	**Is There a Clear Statement of Findings?**	**How Valuable Is the Research?**	**Score**
[35]	Silver Linings	Yes. The aim was to explore positive aspects that are often overlooked in the challenges that mothers of children with ASD face	Yes. Interpretive phenomenological studies are appropriate for exploring the experiences and sense-making of the participants and, therefore, seem well suited for exploring the lived experiences of mothers of children with ASD	Yes. Interviews are suggested to produce in-depth and insightful data	Yes. The research recruited a specific group aligning with the research question. Also, it was claimed to be effective. However, the study excluded fathers, which excludes a perspective that may extend the scope of findings	Yes. It appears that the methods were suitable for capturing the nuanced experiments of participants	Yes. The paper suggests that the researcher was aware of potential impactful factors in the research process	Yes. Ethical approval was given by an institutional board, and details of informed consent were discussed	Yes. The data were thematically analysed, and examples of quotes that drove patterns were shared	Yes. The findings emphasise the emergence of optimism experienced by the mothers in the study. Namely, how hopeful they remained in the face of adversity	(a) This study contributes to the existing knowledge on raising a child with ASD. (b) The study identified the importance of hope and positivity, and (c) further research is needed to explore transerability in contexts and family dynamics	High (12/12)
[36]	The wellbeing of mothers of children with “A-U-T-I-S-M” in Malaysia: An interpretative phenomenological analysis study	Explore the experiences of mothers raising a child with ASD	A qualitative methodology appears appropriate for delving into the richness of human experiences	Yes, a phenomenological approach seems suitable for addressing this question	Yes, the recruitment strategy appears appropriate to the aims; however, excluding two participants due to their form of ASD may have limited the possible depth of the findings	Yes, collecting data from interview transcriptions and IPAs is widely used	The paper could be strengthened by a more explicit discussion of the research–participant relationship	Yes, ethical approval was received from Monash University. While informed consent was not discussed, it could be assumed to be part of the ethical approval	Yes, established methods were employed, and the steps were detailed	Yes, the finding revealed three themes	(a) This research expands the understandings of parenting ASD by focusing on under-represented populations and (b) identifies the strengths of mothers, parenting resilience, and coping strategies. (c) Reseach is needed on how strengths support families in different cultural contexts	Mid (9/12)
[37]	Struggle for recognition, rights, and redistribution: Understanding the identity of parents of children with autism spectrum disorder in China	Explore the identity of parents of children with ASD	Yes, a qualitative approach is well suited to investigating human experiences	Yes, interviews are suggested to produce in-depth and insightful data	Yes, participants were recruited through an organisation that provides therapy to the children	The data collection method chosen is an established way to address the research question	Researchers sought diversity in gender, family income, and family structure, and ensured a comfortable environment for recruitment to reduce any power imbalances. This appears to be evidence of research bias awareness	Yes, ethical approval was granted by the university’s research ethics committee	Yes, researchers mentioned that data collection until saturation was reached, and theme feedback was sought from participants to check for accuracy and strengthen their findings	Three themes were discovered and were supported by quotes from the participants	(a) The study enriches the understanding of family needs and (b) explores coping mechanisms that could enhance parental wellbeing, and may indicate new areas for interventions or support. (c) Research is needed on the effectiveness across cultural setttings	High (12/12)
	**Title**	**Was there a clear statement of the aims** **of the research?**	**Is a qualitative methodology appropriate?**	**Was the research design appropriate to** **address the aims of the research?**	**Was the recruitment strategy appropriate to the aims of the research?**	**Were the data collected in a way that addressed ** **the research issue?**	**Has the relationship between the researcher and participants been adequately considered?**	**Have ethical issues been taken into consideration?**	**Was the data analysis sufficiently rigorous?**	**Is there a clear statement of findings?**	**How valuable is the research?**	**Score**
[38]	Exploring fathers’ perceptions of parenting a child with Asperger syndrome	The aims were to understand the perspectives of fathers of children with ASD and provide insights into the challenges and rewards of parenting a child with ASD	Yes, a qualitative methodology seems appropriate for exploring human experiences	Yes, a phenomenological approach seems suitable for exploring the perceptions of fathers of children with ASD	Yes, the sample of fathers of children with ASD was recruited from rural and urban areas. However, the sample size was small, *n* = 7, and excluded mothers; this excludes a perspective that may extend the scope of findings	Yes, semistructured interviews are suggested to be suitable for qualitative research. However, bias is a possibility due to the past relationship between the interviewer and some participants	Interviewer relations with participants are a point of concern and possible bias.	The clinical research ethics committee granted ethical approval at a university hospital. In addition, informed consent and confidentiality were explored	The Colaizzi method used in this study’s results is generally considered trustworthy and credible	The findings named positive aspects such as discovering a child’s positive traits, pride in seeing the child achieving milestones, and enjoying signs of affection, such as hugs	(a) Contributes to the understanding of the experiences of children with ASD. (b) Highlights the importance of considering the positive aspects of parenting experiences. (c) Research is needed to evaluate how positive experiences can be supported in diverse family settings	Mid (9/12)
[39]	“We are in this together”: Experiences of relationship satisfaction in couples raising a child with autism spectrum disorder	Explore the experiences of parents of children with ASD to understand relationship satisfaction between parents	Yes, a qualitative methodology seems appropriate for exploring human experiences	Yes, in-depth interviews followed by IPAs seem appropriate as they allow for the exploration of the participants’ lived experiences	The recruitment was discussed, and participants were recruited from a previous survey	Semistructured interviews appear to be a well-suited method to collect data to address the research question	Researchers ensured that informed consent forms were complete and participants understood their rights. Researchers also sought participant feedback on themes that reflected their shared experiences	The study received ethics approval from the Curtin University Human Research Ethics Committee	Established methods were employed by a research team; data collection reached saturation. Therefore, no additional themes were expected after this point. Participant feedback on themes increased the strength of the accuracy of the findings	Three themes emerged: shared beliefs, teamwork, and shared experiences	(a) The study reinforces the importance of family resiliences with ASD, and (b) highlights the need to support and promote resilience. (c) Research is needed to determine the most effective strategies that maintain resilience in families with a child with ASD	High (12/12)
[40]	The resilience of parents who have children with autism spectrum disorder in China: a social culture perspective	Yes, the aim is to explore how parents develop resilience in China and discover the characteristics of their resilience	As an under-researched topic, designing a qualitative approach seems appropriate for understanding this topic more deeply and generating new and rich data	Yes; to go deep, a qualitative design was needed. It is also flexible and adaptive to a changing theme	Yes, the study achieved samples of parents who met the criteria	Yes, the interviews used are believed to allow for in-depth insights into the thoughts and experiences of parents	Yes, the research involved activities that were believed to build trust with participants	The study sought ethical approval from the university. Confidentiality was maintained	The data analysis programme NVivo 8 was used, and applied social ecosystems theory was used to guide the analysis and identify themes	The findings were that parents cope with stress by reflecting on themselves to search for personal growth. Parents read about moral principles from traditional texts. Support networks are important	(a) The study offers insights into the positive aspects of parenting a child with ASD and (b) may open new avenues for research. (c) Research is needed to generalise findings across diverse contexts	High (12/12)
